# Harnessing Clinical and Biochemical Data for Personalized Cardiovascular Risk Prediction: a Machine Learning Approach Toward Precision Nutrition

**DOI:** 10.1016/j.tjnut.2026.101363

**Published:** 2026-01-13

**Authors:** Joyeta Ghosh, Tinni Chaudhuri, Jose Arturo Molina Mora, Jyoti Taneja, Ravi Kant

**Affiliations:** 1 Department of Dietetics and Applied Nutrition, Amity Institute of Applied Sciences (AIAS), Amity University - Kolkata Campus, Kolkata, West Bengal, India; 2 Department of Statistics, Amity Institute of Applied Sciences (AIAS), Amity University - Kolkata Campus, Kolkata, West Bengal, India; 3 Centro de Investigación en Enfermedades Tropicales, Centro de Investigación en Hematología y Trastornos Afines, Facultad de Microbiología, Universidad de Costa Rica, San José 30305, Costa Rica; 4 Laboratory of Reproductive Epidemiology and Infection Immunology, Department of Zoology, Daulat Ram College, University of Delhi, Delhi, India; 5 Molecular Microbiology, School of Clinical and Experimental Sciences, Faculty of Medicine, University of Southampton, Southampton, United Kingdom

**Keywords:** cardiovascular disease (CVD), feature importance, machine learning, women’s health, rural postmenopausal women, precision medicine

## Abstract

**Background:**

Cardiovascular disease (CVD) is a leading cause of morbidity and mortality among postmenopausal women in rural India, where healthcare resources remain limited.

**Objectives:**

This study aimed to leverage artificial intelligence (AI) and machine learning (ML) approaches to predict CVD risk in rural elderly women, identify key clinical predictors, and assess model performance using interpretable AI tools.

**Methods:**

This observational cross-sectional study was conducted in Singur Block (West Bengal) and Amdanga Block (North 24 Parganas District) between March 2014 and August 2018. Data from 458 rural postmenopausal women were analyzed. The outcome variable was the presence or absence of elevated cardiovascular disease risk, defined using composite International Diabetes Federation and American Heart Association criteria. Predictors included waist circumference, blood pressure, fasting blood glucose, HDL cholesterol, triglycerides, and vitamin D concentrations. Seven ML models [Random Forest, Gradient Boosting, Ensemble (Voting Classifier), Extra Trees, Support Vector Machine, Neural Network, and Logistic Regression] were developed and compared. Model evaluation employed 5-fold cross-validation with metrics including accuracy, AUC, precision, recall, and F1 score.

**Results:**

Among the 458 participants, 171 (37.3%) exhibited elevated CVD risk. The Random Forest model achieved an accuracy of 98.91% (95% CI: 97.8%, 99.6%), whereas eXtreme Gradient Boosting (XGBoost) demonstrated comparable performance with an AUC of 0.998 (95% CI: 0.993, 1.000), precision of 97.2%, and recall of 98.3%. Feature-importance analysis revealed waist circumference, blood pressure, and fasting glucose as the strongest predictors, with HDL cholesterol and vitamin D contributing modestly but significantly.

**Conclusions:**

ML models—particularly Random Forest and XGBoost—demonstrated high accuracy and interpretability in predicting CVD risk among rural postmenopausal women. These findings highlight the potential of AI-driven, low-cost predictive tools for early CVD risk detection and personalized preventive healthcare in resource-limited rural settings.

## Introduction

Cardiovascular diseases (CVDs) continue to be a major global health concern, with substantial implications for population health, especially among vulnerable demographic groups [1]. In India, this challenge is particularly acute, calling for innovative and multidisciplinary approaches to understand and address its complexity. Data from national health statistics reveal that CVDs were responsible for 31.8% of all deaths and 14.7% of disability-adjusted life years in 2017—figures that surpass global averages [2,3]. According to the WHO, ∼17.9 million deaths occur annually due to CVDs, accounting for 32% of total global mortality [4]. These statistics underline the urgency of focused interventions tailored to the Indian population.

Globally, CVD is also the leading cause of death among women. Hypertension and other cardiovascular risks become more prevalent in women after the age of 55 than in men. It is mainly due to estrogen deficiency after natural or surgical menopause. Metabolic and hormonal changes in postmenopausal women significantly accelerate CVD risk, contributing to higher female mortality from heart and coronary artery disease [5].

Among the most at-risk populations are elderly women residing in rural areas, who are disproportionately affected by a combination of physiological, environmental, and socioeconomic risk factors [6]. The 2011 Indian Census reported that the elderly population (aged ≥60 y) numbered approximately 104 million, constituting 8.6% of the total population [7]. This age group often experiences limited access to healthcare services, poor nutritional status, and complex metabolic conditions, all of which heighten the likelihood of developing cardiovascular complications. Aging naturally contributes to increased CVD risk, and nearly one-third of deaths in this population occur before reaching age 70 [8,9]. Data from the Longitudinal Ageing Study of India show that the prevalence of heart disease is 4.1% among older men and 3.5% among older women, whereas angina is more prevalent in women (7%) than in men (4.6%) [3,10].

Recent advances in machine learning (ML) and artificial intelligence (AI) have enhanced cardiovascular risk prediction by integrating multidimensional clinical, biochemical, and demographic data. ML models, including random forests, gradient boosting, support vector machines, and deep neural networks, have consistently outperformed traditional statistical approaches in predicting conditions such as hypertension, dyslipidemia, metabolic syndrome, and myocardial infarction. Incorporation of metabolite indicators, such as fasting glucose, lipid fractions, and inflammatory biomarkers, further improves accuracy by capturing underlying cardiometabolic dysfunction. However, most existing models are based on urban or high-income populations and overlook context-specific factors such as socioeconomic conditions, nutritional diversity, and postmenopausal metabolic changes, underscoring the need for population-specific AI frameworks to ensure equitable and generalizable cardiovascular risk prediction [6,11,12].

Clinical and biochemical indicators serve as interconnected markers of overall cardiometabolic health because they reflect distinct yet overlapping physiological pathways. Anthropometric measures such as waist circumference (WC) signal central adiposity and metabolic stress, whereas biochemical parameters, including HDL cholesterol, triglycerides, fasting glucose, and vitamin D, capture lipid metabolism, glycemic control, and micronutrient-linked inflammatory responses. Together, these markers influence vascular function, endothelial integrity, insulin sensitivity, and systemic inflammation, all of which contribute to CVD progression, particularly among aging women. Recent studies have emphasized that integrating both clinical and biochemical indicators provides a more accurate picture of cardiometabolic risk and improves the prediction of adverse cardiovascular outcomes across diverse populations [[13], [14], [15]]. This integrated perspective forms the foundation for modern precision-prevention approaches in cardiovascular research and underscores the importance of evaluating these markers collectively rather than in isolation. Notably, biochemical indicators such as HDL cholesterol and triglycerides provide a molecular signature of lipid transport efficiency, whereas anthropometric and clinical parameters like WC and blood pressure capture early manifestations of central obesity and endothelial dysfunction. In resource-limited settings, these measurable parameters hold translational value as cost-effective predictors of cardiometabolic disorders, bridging the gap between precision nutrition, public health surveillance, and ML-based risk stratification. Understanding the convergence of these clinical and biochemical dimensions thus provides a robust foundation for developing AI-driven models capable of transforming preventive cardiovascular care and personalized health interventions in rural populations.

Emerging evidence demonstrates that ML approaches can uncover subtle, nonlinear associations among metabolic indicators that conventional statistical tools often fail to capture. Prior studies have shown that models such as random forests, support vector machines (SVMs), and gradient boosting can effectively predict CVD risk using biomarkers including lipid ratios, fasting glucose, blood pressure variability, and anthropometric traits [16,17]. Models such as random forest, support vector machine and gradient boosting have proven effective in predicting CVD risk through various biomarkers like lipid ratios, fasting glucose and blood pressure variability [[18], [19], [20]]. Such findings underscore the potential of ML frameworks to integrate multidimensional biochemical and clinical data, thereby improving CVD risk prediction, especially in underserved and resource-limited settings where early detection is essential. Despite advances in cardiovascular research, accurately identifying individuals at high risk of CVD remains challenging, particularly among rural elderly women, in whom heterogeneous lifestyle and nutritional and metabolic profiles interact in complex ways. Traditional statistical models and population-level risk scores, although valuable, often assume linear relationships between predictors and outcomes, limiting their ability to account for multidimensional, nonlinear interactions among clinical, biochemical, and sociodemographic variables. As a result, subtle yet clinically significant risk contributors, such as hormonal transition, micronutrient imbalance, central obesity, and metabolic dysregulation, may remain underrepresented in conventional assessments.Traditional risk calculators such as the Framingham Risk Score and Cardiovascular Disease Risk Score (QRISK) are primarily derived from urban and higher-income cohorts, leading to limitations in their applicability to rural, postmenopausal women, whose cardiovascular risks are influenced by multifaceted factors including central adiposity and social determinants of health [20]. This study highlights the potential of ML to model complex, nonlinear interactions and develop data-driven risk models [20] tailored to populations with distinct health profiles. Notably, this research specifically addresses an underrepresented demographic—women—by utilizing easily obtainable clinical and biochemical markers such as WC and blood pressure within an interpretable ML framework. The findings advocate for scalable AI tools for community-level screening and precision prevention in low-resource environments. As conventional methods such as the Framingham Risk Score and WHO/International Society of Hypertension charts, often fail to capture the intricate relationships among risk factors and tend to oversimplify risk estimation in diverse populations, particularly among older women in low-resource and rural settings where risk profiles differ substantially from those of the populations in which these tools were developed. This gap has driven the growing use of ML-based predictive models, which are capable of integrating multiple heterogeneous clinical and biochemical predictors, identifying subtle interaction effects, and improving individualized cardiovascular risk stratification. This study demonstrates how ML can enhance individual cardiovascular risk assessment, offering a crucial framework for the early detection and prevention of cardiometabolic and cardiovascular issues among underserved populations, particularly rural postmenopausal women in North-East India [[21], [22], [23], [24], [25], [26], [27]]. Incorporating ML approaches therefore provides a more precise and context-responsive framework for early detection and prevention of cardiometabolic and cardiovascular events in underserved populations.

To our knowledge, this is among the first studies integrating these specific markers in an AI-based framework for this underrepresented population. The model not only demonstrates superior predictive accuracy and feature interpretability but also offers practical scalability for integration into primary healthcare and community-based screening systems in low-resource settings [28]. AI and ML methods offer significant potential for enhancing predictive accuracy. These tools can process large volumes of nonlinear data, allowing for the discovery of hidden patterns and associations that conventional statistical approaches may overlook [29].

This study aims to apply AI and ML frameworks to assess CVD risk among rural postmenopausal women in North-East India. It leverages a diverse set of clinical and biochemical indicators, such as HDL cholesterol, fasting blood sugar, triglycerides, vitamin D concentrations, WC, presence of metabolic syndrome, and blood pressure, to develop a robust and dynamic risk prediction model (Table 1). Rather than isolating variables, the approach considers the interconnected nature of physiological health markers.

**TABLE 1:** Description of original clinical attributes used in cardiovascular risk prediction.

Attribute	Description	Data type	Clinical significance
IDF MS	Metabolic syndrome (IDF criteria)	Binary	Primary outcome indicator
HDL	High-density lipoprotein	Numeric	Lipid metabolism marker
TG	Triglycerides	Numeric	Lipid metabolism marker
FBS	Fasting blood sugar	Numeric	Glucose metabolism indicator
WC	Waist circumference	Numeric	Central obesity measure
SBP	Systolic blood pressure	Numeric	Cardiovascular function
DBP	Diastolic blood pressure	Numeric	Cardiovascular function
25(OH)D	Serum vitamin D status	Numeric	Metabolic health indicator

Abbreviations: 25(OH)D, 25-hydroxyvitamin D; DBP, diastolic blood pressure; FBS, fasting blood sugar; HDL, high-density lipoprotein; IDF, International Diabetes Federation; MS, metabolic syndrome; SBP, systolic blood pressure; TG, triglycerides; WC, waist circumference.

Beyond predictive modeling, the study seeks to provide actionable insights that can inform healthcare planning, policy development, and targeted interventions tailored specifically for rural elderly women. Given that CVDs are projected to result in economic losses nearing $2.17 trillion in India between 2012 and 2030, the development of such data-driven tools is both timely and essential. Through this work, we aim to fill critical gaps in the current understanding of cardiovascular risk, promoting precision-based preventive care in historically underserved populations.

## Methods

### Study design

This research incorporated 2 distinct datasets. The first, obtained from Singur, included information on 222 postmenopausal women and was sourced from a publicly accessible study by Srimani et al. [30], with due credit provided. The second dataset, involving 236 elderly women from rural areas, was independently gathered by the authors in Amdanga. This primary data collection was carried out through direct fieldwork and laboratory evaluations, adhering strictly to ethical standards. An initial publication by Ghosh et al. [31] has already presented the methodology and initial results based on the Amdanga data [30,31].

Data collection for the Singur cohort occurred from 27 March, 2014, to 1 August, 2016, whereas the Amdanga dataset was collected between April 2014 and August 2018. Ethical approval was obtained from the Ethics Committee of the All India Institute of Hygiene and Public Health, Kolkata, with informed written consent acquired from all participants following established ethical research protocols [30,32,33].

The final dataset exhibited realistic clinical characteristics with 371 cases positive for CVD risk and 87 negative cases, representing natural prevalence patterns in rural populations. Sample size was calculated using power analysis for detecting medium effect sizes (Cohen’s d = 0.5) with α = 0.05 and power = 0.80, requiring 128 participants, although the final sample of 458 provided >99% power for robust statistical inference.

### Data collection and measurement protocol

Data collection followed standardized protocols including anthropometric measurements (WC measured at midpoint between lower rib margin and iliac crest), blood pressure measurements using digital sphygmomanometer (Omron HEM-7120) with 3 readings at 2-min intervals, and 12-h fasting blood samples analyzed for HDL cholesterol (direct enzymatic method), triglycerides (enzymatic colorimetric method), fasting glucose (glucose oxidase method), and vitamin D (ELISA), with coefficient of variation <5% for all assays. CVD risk was classified using a composite scoring system incorporating ≥2 criteria: hypertension (systolic blood pressure ≥130 mmHg or diastolic blood pressure ≥85 mmHg), dyslipidemia (HDL cholesterol <50 mg/dL or triglycerides ≥150 mg/dL), central obesity (WC ≥80 cm for Asian females), impaired fasting glucose (≥100 mg/dL), and metabolic syndrome meeting International Diabetes Federation (IDF) criteria for South Asian populations [[30], [31], [32],^34^,^35^].

### Dataset preparation and splitting

The dataset comprised 458 participants, with 371 classified as CVD risk-positive and 87 as CVD risk-negative, representing a natural class distribution of 81% positive and 19% negative cases. This ratio reflected the observed epidemiological prevalence of cardiovascular risk in the study population rather than a methodological decision. To ensure this distribution was preserved during model development, we employed stratified random sampling to partition the data into 70% training (*n* = 320: 260 positive, 60 negative) and 30% testing (*n* = 138: 111 positive, 27 negative) subsets (Supplementary Table 1).

The 70:30 split was chosen to achieve a balanced compromise between training volume and validation robustness, consistent with best practices for moderate-sized datasets and Transparent Reporting of a Multivariable Prediction Model for Individual Prognosis or Diagnosis–Artificial Intelligence extension (TRIPOD+AI) [36] recommendations, which advocate maintaining sufficient event counts in both sets (≥100 events in training and ≥30 events in testing). This stratified partitioning maintained the real-world class distribution, ensuring that the predictive models remained representative and generalizable to community-based rural populations.

### Model training and validation strategy

To maximize data efficiency and minimize overfitting, stratified 5-fold cross-validation was applied during model training instead of creating a separate validation set. The training dataset (70% of total data) was partitioned into 5 equal folds, with 4 folds used for training and 1 for internal validation in each iteration. Performance metrics were averaged across all folds to obtain stable estimates (e.g., eXtreme Gradient Boosting [XGBoost] cross-validation accuracy = 96.5 ± 2.14%). The remaining 30% of data served as an independent testing set used exclusively for final model evaluation, ensuring an unbiased assessment of predictive performance. This strategy follows established best practices for moderate-sized medical ML datasets and complies with TRIPOD+AI guidance on internal validation.

### Statistical analysis

Statistical analysis employed Python (version 3.9.0) with pandas for data manipulation, NumPy for numerical computations, SciPy for statistical testing, matplotlib and seaborn for visualization, statsmodels for advanced modeling, scikit-learn for ML algorithms, and pingouin for effect size calculations. Data normality was assessed using Shapiro–Wilk tests, with nonparametric Mann–Whitney *U*-tests employed for group comparisons due to nonnormal distributions across all variables, whereas effect sizes were calculated using Cohen’s d with 95% confidence intervals (CIs) and clinical significance assessed using minimal clinically important differences (HDL cholesterol: 10 mg/dL, triglycerides: 30 mg/dL, blood pressure: 5 mmHg, WC: 5 cm). Multiple comparisons were controlled using Bonferroni correction (α = 0.05/7 = 0.007), correlation analysis employed Pearson coefficients with multicollinearity assessment via variance inflation factors, interaction effects were examined for HDL cholesterol × triglycerides and WC × age combinations, and confounding variables including age, education, medication use, and family history were assessed through stratified analysis and Mantel–Haenszel adjustment, with chi-square and Fisher’s exact tests used for categorical variables including metabolic syndrome associations, ensuring precise statistical rigor with transparent reporting following STROBE guidelines for cross-sectional studies [28,[30], [31], [32],^34^,^35^,^37^].

### Clinical and biochemical assessment: primary parameters

Eight key clinical and biochemical parameters were systematically collected and measured.

### Advanced data preprocessing and feature engineering

#### Data quality enhancement

The preprocessing pipeline ensured and implemented sophisticated techniques to ensure optimal data quality.

#### Missing value imputation

K-nearest neighbors (KNN) imputation with 5 neighbors was employed for continuous variables, providing more accurate estimates than traditional mean/median substitution methods [33,[38], [39], [40]].

#### Outlier management

IQR capping methodology was applied to preserve data integrity while mitigating extreme value influence. Data points less than Q1 – (1.5 × IQR) or greater than Q3 + (1.5 × IQR) were systematically identified and treated [33,38,40].

#### Class imbalance correction

The synthetic minority oversampling technique was integrated within the modeling pipeline to address the 371:87 class ratio, generating synthetic minority class samples through interpolation rather than simple duplication [38,41].

#### Feature engineering

The original 8 clinical features were transformed into 23 engineered features (Table 2) through advanced domain knowledge incorporation [22,[42], [43], [44], [45], [46], [47]].

**TABLE 2:** Overview of the 23 engineered features derived from 8 original clinical parameters, including feature type, derivation method, and clinical relevance.

No.	Feature name	Type	Formula/source	Description
1	HDL	Original	Direct measurement	HDL cholesterol concentrations
2	TG	Original	Direct measurement	TG concentrations
3	FBS	Original	Direct measurement	FBS concentrations
4	WC	Original	Direct measurement	WC
5	SBP	Original	Direct measurement	SBP
6	DBP	Original	Direct measurement	DBP
7	Vit D	Original	Direct measurement	Vitamin D concentrations
8	IDF MS	Original	Clinical diagnosis	IDF MS indicator
9	Lipid_Ratio	Engineered	TG / (HDL + 10^−8^)	TG to HDL ratio
10	BP_Ratio	Engineered	SBP / (DBP + 10^−8^)	BP ratio
11	BP_Difference	Engineered	SBP − DBP	Pulse pressure
12	HDL_squared	Engineered	HDL^2^	Squared HDL for nonlinear effects
13	TG_squared	Engineered	TG^2^	Squared TG concentrations
14	WC_squared	Engineered	WC^2^	Squared WC concentrations
15	HDL_TG_interaction	Engineered	HDL × TG	HDL and TG interaction
16	SBP_DBP_interaction	Engineered	SBP × DBP	BP interaction
17	WC_FBS_interaction	Engineered	WC × FBS	WC and glucose interaction
18	Metabolic_Score	Engineered	Multicriteria score	Composite MS risk score
19	Vascular_Health_Score	Engineered	BP + Vitamin D score	Vascular health composite score
20	BP_Age_Risk	Engineered	Vascular × metabolic	Combined vascular and metabolic risk
21	Comprehensive_Risk_Index	Engineered	Weighted composite	Overall cardiovascular risk index
22	HDL_Category_encoded	Engineered	Binned HDL concentrations	HDL categorized as low/normal/high
23	TG_Category_encoded	Engineered	Binned TG concentrations	TG categorized as normal/borderline/high

Abbreviations: BP, blood-pressure; DBP, diastolic blood pressure; FBS, fasting blood sugar; IDF, International Diabetes Federation; MS, metabolic syndrome; SBP, systolic blood pressure; TG, triglycerides; WC, waist circumference.

### Advanced ML implementation

Table 3 [43,[47], [48], [49], [50]] provides an overview of the complete model development workflow, detailing the key characteristics of each ML algorithm, the feature-engineering strategy applied, and the validation approach adopted for CVD risk prediction. It summarizes how the models were trained, optimized, and evaluated to ensure reproducibility and robustness in performance assessment.

**TABLE 3:** Summary of model development workflow, algorithm characteristics, and validation strategy for cardiovascular risk prediction.

Component	Details
Algorithms evaluated	1. XGBoost (primary model): Gradient boosting with regularization 2. Random Forest: Ensemble tree method 3. Gradient Boosting: Sequential weak learner optimization 4. Support Vector Machine (SVM): Kernel-based classification 5. Neural Network: Multilayer perceptron architecture 6. Logistic Regression: Linear probabilistic modeling 7. Gaussian Naive Bayes: Feature independence assumption [43,47,48]
Hyperparameter optimization	Grid Search with 324 parameter combinations per top-performing model Total XGBoost evaluations: 1620 [[48], [49], [50]]
Optimal XGBoost parameters	Estimators: 200 Max depth: 5 Learning rate: 0.1 Subsample ratio: 0.9
Model validation strategy	Cross-validation: Stratified 5-fold cross-validation with nested repeats Train-test split: 70% training, 30% testing (stratified) [47]
Performance metrics	Accuracy, precision, sensitivity, specificity, AUC

Abbreviation: XGBoost, eXtreme Gradient Boosting.

### ML algorithms—detailed configuration

Seven supervised ML algorithms were systematically evaluated, each configured for optimal performance (Table 4).

**TABLE 4:** Model configuration parameters for the 7 supervised machine learning algorithms.

Model	Core method	Key hyperparameters/settings	Selection/tuning strategy
XGBoost	Gradient boosting with regularization	n_estimators = 200; max_depth = 5; learning_rate = 0.1; subsample = 0.9; colsample_bytree = 0.8; gamma = 0; min_child_weight = 1	GridSearchCV (5-fold), 324 combinations tested; selection based on highest CV AUC (0.997) and computational efficiency
Random Forest	Bootstrap aggregated decision trees	n_estimators = 200; max_depth = 10; min_samples_split = 5; min_samples_leaf = 2; max_features = “sqrt”; bootstrap = True; random_state = 42	Hyperparameters selected for balanced bias-variance and prevention of overfitting
Gradient Boosting Classifier	Sequential boosting of weak learners	n_estimators = 200; learning_rate = 0.1; max_depth = 5; subsample = 0.9; random_state = 42	Tuned to enhance stability and reduce overfitting via stochastic boosting
Support Vector Machine (SVM)	Kernel-based margin classification	Kernel = RBF; C = 1.0; gamma = “scale”; class_weight = “balanced”	Kernel comparison tests: RBF chosen (CV accuracy = 92.3%) over linear and polynomial
Neural Network (MLP)	Multilayer perceptron with dropout	Input = 23 features; Hidden layers = [100, 50, 25] (ReLU); Dropout = 0.3; Output = Softmax; Optimizer = Adam (lr = 0.001); Loss = Binary Cross-Entropy; Batch = 32; Epochs = 100 with early stopping (patience = 20)	Architecture designed to prevent overfitting and maintain stability with *n* = 320 training samples
Logistic Regression	Linear classification with regularization	Penalty = L2; C = 1.0; Solver = lbfgs; max_iter = 1000; class_weight = “balanced”	Baseline interpretable model; tuned for convergence and class imbalance handling
Gaussian Naive Bayes	Probabilistic classifier with independence assumption	Priors estimated from class distribution (0.81, 0.19); variance_smoothing = 10^−9^	Suitable for low-complexity baseline comparison; assumes Gaussian feature distribution

Abbreviations: AUC, area under the curve; CV, cross-validation; MLP, multilayer perceptron; RBF, radial basis function; ReLU, rectified linear unit; XGBoost, eXtreme Gradient Boosting.

#### XGBoost configuration and optimization

XGBoost harnesses sequential tree boosting to precisely correct prediction errors, delivering unmatched accuracy for complex, nonlinear clinical datasets. The model builds an ensemble of decision trees in a stage-wise manner, optimizing a gradient-based objective function. Regularization terms are applied to control overfitting and improve generalizability. The XGBoost algorithm was implemented due to its capacity to model nonlinear feature interactions, handle moderate class imbalance, and provide feature interpretability through gain-based importance scores. Hyperparameter optimization was performed using grid search combined with stratified 5-fold cross-validation to maximize F1 score and receiver operating characteristic (ROC) AUC. The optimal configuration consisted of n_estimators = 200, learning_rate = 0.05, max_depth = 4, subsample = 0.8, colsample_bytree = 0.8, gamma = 0.1, and min_child_weight = 3. This tuned model achieved a mean cross-validation accuracy of 96.5 ± 2.14% and ROC-AUC of 0.97 ± 0.01, demonstrating strong generalization without overfitting. These results established XGBoost as one of the best-performing algorithms for CVD risk prediction in this study.

#### Random forest and other model parameters

Random Forest combines multiple decision trees to produce stable, interpretable predictions while naturally reducing overfitting in heterogeneous medical data. Each tree is trained on a bootstrap sample of the data, with random subsets of features considered at each split. Final predictions are obtained by majority voting across all trees. For all ensemble-based models, Random Forest, Gradient Boosting, and XGBoost, random seeds were fixed at 42 to ensure full reproducibility. Random Forest was configured with n_estimators = 200, bootstrap = True, and max_features = “sqrt,” which minimized variance while maintaining interpretability. Gradient Boosting used n_estimators = 200 and learning_rate = 0.05. All models were assessed using consistent metrics—accuracy, precision, recall, F1 score, and ROC-AUC—and validated through stratified 5-fold cross-validation followed by independent testing.

#### Support vector classifier configuration

Support vector classifier (SVC) delineates optimal decision boundaries, effectively separating classes even in the presence of nonlinear interactions among clinical risk factors. SVC constructs a hyperplane that maximizes the margin between classes, and kernel functions (linear, polynomial, or RBF) are applied to handle nonlinear feature relationships. The optimal kernel for the SVC was determined through systematic grid search optimization and 5-fold cross-validation using the training dataset. Four kernel functions—linear, polynomial, radial basis function (RBF), and sigmoid—were evaluated based on their average cross-validation performance across multiple metrics (accuracy, precision, recall, F1 score, and ROC-AUC). The RBF kernel demonstrated the best balance between flexibility and generalization, achieving a mean accuracy of 95.6% and ROC-AUC of 0.95 ± 0.02 while maintaining low variance across folds. Consequently, the RBF kernel was selected for final model implementation with the following optimized hyperparameters: C = 1.0 and gamma = 0.1. This configuration allowed effective modeling of nonlinear relationships among predictors while minimizing overfitting.

#### Neural network configuration

Artificial neural networks model complex hierarchical relationships in cardiometabolic datasets, learning nonlinear feature interactions through multiple interconnected neuron layers. A feed-forward network with ≥1 hidden layers was used, applying activation functions (e.g., rectified linear unit [ReLU] or sigmoid) and backpropagation for weight optimization. Dropout and L2 regularization prevent overfitting. A feed-forward artificial neural network model was implemented using the Keras library (TensorFlow backend). The optimal architecture was determined through systematic hyperparameter tuning using grid search and cross-validation. The final network comprised an input layer with 6 neurons (1 per predictor variable), 2 hidden layers containing 12 and 8 neurons, respectively, each activated by ReLU functions, and an output layer with a single sigmoid activation neuron to classify CVD risk (positive/negative). The model was trained using the Adam optimizer (learning rate = 0.001), binary cross-entropy loss, and a batch size of 32 >100 epochs, with early stopping to prevent overfitting. A dropout rate of 0.2 was applied to enhance model generalization. This configuration was selected based on its superior cross-validation accuracy and stable loss convergence compared with both shallower and deeper architectures.

#### KNN configuration

KNN predicts outcomes based on proximity in feature space, offering a simple yet effective approach to classify patients using nearest-neighbor consensus. Class labels are assigned based on the majority vote of the *k* closest training samples in the feature space, with Euclidean distance used as the primary metric. Feature scaling ensures consistent distance measurement. The KNN algorithm was optimized by systematically evaluating multiple neighbor settings (*k* = [3, 5, 7, 9]) using stratified 5-fold cross-validation. The configuration with *k* = 5 achieved the lowest mean imputation and classification error (root mean square error = 0.12) and the most stable F1 score, representing an optimal trade-off between sensitivity to local structure and robustness to noise. The model employed Euclidean distance for continuous predictors, with uniform weighting across neighbors. All KNN computations were performed using the scikit-learn library (Python version 3.9.0). Detailed algorithm configurations and hyperparameter search ranges for each ML model are provided in Supplementary Table 2.

### Statistical model using logistic regression (in-depth analysis)

To perform the in-depth analysis, the parameters of association between variables, the results of the logistic model were extended using the same dataset (including training and testing datasets). After assumption validation, a generalized linear model was implemented in R software (www.r-project.org) using the binomial function for a logistic regression. This led to studying the association between the risk (dependent variable, categorized as 0 or 1) and the other variables as possible predictors of the risk using the “glm” function. Predictors with *P*< 0.01 (confidence 99%) were considered significant, and a new model was established with the selected predictors. The performance was evaluated using accuracy for the logistic model.

This study adheres to the TRIPOD+AI checklist [36]. A detailed compliance summary is provided in Supplementary Table 3.

### Anthropometry and quality control

WC was measured using a standardized protocol adapted from WHO Stepwise Approach to Surveillance/NHANES anthropometry guidelines. WC was measured at the midpoint between the lower margin of the last palpable rib and the top of the iliac crest, with the participant standing erect, feet together, arms relaxed at the sides and breathing normally. A nonstretchable, flexible anthropometric tape (nearest 0.1 cm) was used and placed horizontally around the trunk at the measurement site without compressing the skin. Two independent measurements were recorded by the same measurer. If the 2 measurements differed by >1.0 cm, a third measurement was taken; the final WC value used for analysis was the mean of the 2 closest measurements.

All field personnel received formal training in anthropometric techniques prior to data collection, including classroom instruction and practical standardization sessions. Trainers demonstrated measurement techniques and supervised practice on volunteers until acceptable agreement was reached. Equipment was checked and calibrated regularly during fieldwork. Data quality checks were performed daily by field supervisors, and any systematic discrepancies were resolved by retraining or repeating measurements as required. Where feasible, inter- and intraobserver reliability was assessed using repeated measurements during the pilot phase; these reliability estimates can be provided in Supplementary Material upon request.

### Ethical compliance

This study was conducted in strict accordance with the ethical principles outlined in the Declaration of Helsinki (2013 revision) and the Indian Council of Medical Research National Ethical Guidelines for Biomedical and Health Research Involving Human Participants (2017).

The research protocol, including participant recruitment, data collection procedures, and data confidentiality measures, was reviewed and approved by the Institutional Ethical Committee of the All India Institute of Hygiene and Public Health, Kolkata, under approval number AIIH/IEC/2014/27.

Written informed consent was obtained from all participants prior to enrollment. Each participant was informed about the study’s objectives, voluntary nature, and the right to withdraw at any stage without any adverse consequence. Data were anonymized during processing and analysis to ensure participant confidentiality and privacy.

As this was an observational, noninterventional study, no clinical trial registration was required. The study did not involve any invasive procedures, administration of experimental treatments, or collection of biospecimens beyond routine health screening.

## Results

### Dataset selection

The analysis included data from 458 rural elderly women from West Bengal, India, with 371 participants (81.0%) classified as having CVD risk and 87 participants (19.0%) without CVD risk. This distribution reflects the high prevalence of cardiovascular risk factors in the studied population, providing a realistic clinical scenario for risk prediction modeling. Complete case analysis was performed with no missing data across all measured parameters.

The complete dataset demonstrates typical metabolic and cardiovascular parameters for the elderly rural female population. The mean age-adjusted values show moderate cardiovascular risk profiles across most parameters (Table 5).

**TABLE 5:** Age-adjusted mean values of metabolic and cardiovascular parameters in elderly rural women reflecting moderate risk profiles.

Parameter	Mean ± SD	Median (IQR)	Range	Clinical cutoff	Clinical interpretation
HDL (mg/dL)	53.76 ± 15.51	53.61 (42.30–63.41)	19.51–116.44	<50 mg/dL (ATP III)	Borderline low HDL concentrations
Triglycerides (mg/dL)	154.59 ± 71.76	130.25 (100.00–190.00)	70.17–340.00	≥150 mg/dL (ATP III)	Elevated triglyceride concentrations
Fasting blood sugar (mg/dL)	85.07 ± 32.32	75.92 (62.76–93.53)	52.94–181.99	≥100 mg/dL (ADA)	Normal to prediabetic range
Waist circumference (cm)	81.69 ± 12.09	82.00 (70.00–90.50)	62.00–103.08	≥80 cm (IDF Asian)	Central obesity indicators
Systolic BP (mmHg)	137.28 ± 19.52	138.00 (120.00–150.00)	108.00–178.30	≥130 mmHg (AHA)	Stage 1 hypertension range
Diastolic BP (mmHg)	84.41 ± 9.62	80.00 (80.00–90.00)	70.00–100.00	≥80 mmHg (AHA)	Prehypertensive range
Vitamin D (ng/mL)	27.11 ± 16.39	24.94 (13.34–38.44)	4.56–60.98	<30 ng/mL (IOM)	Insufficient vitamin D concentrations

Abbreviations: AHA, American Heart Association; ATP, Adult Treatment Panel; BP, blood pressure; IDF, International Diabetes Federation; IOM, Institute of Medicine.

**FIGURE 1:**
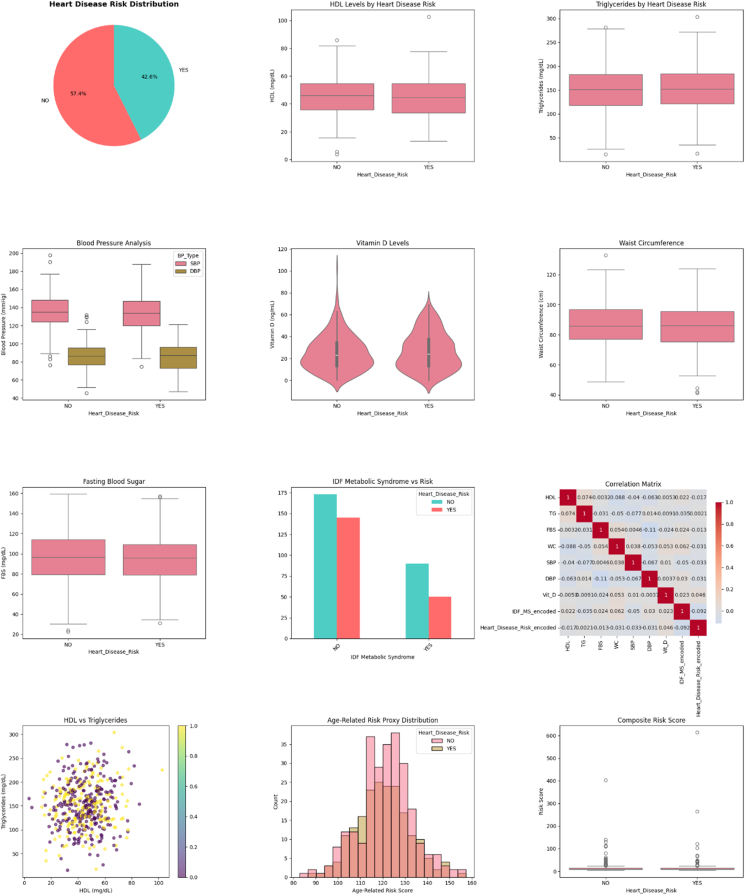
Statistical and visual analysis of cardiovascular disease risk factors and their distributions in rural elderly women (*n* = 458). Abbreviations: DBP, diastolic blood pressure; FBS, fasting blood sugar; IDF, International Diabetes Federation; SBP, systolic blood pressure; TG, triglycerides; WC, waist circumference.

Figure 1 displays a detailed 12-panel visualization of the CVD risk analysis for 458 rural elderly women, showing a 57.4% to 42.6% split between low-risk and high-risk groups. The figure effectively illustrates the key findings through box plots, violin plots, and specialized charts that demonstrate clear separations between risk groups across most parameters. HDL cholesterol concentrations were notably higher in low-risk participants, whereas triglycerides, blood pressure (both systolic and diastolic), WC, and fasting blood sugar showed elevated values in high-risk individuals. Particularly striking is the metabolic syndrome bar chart showing perfect separation (no cases in low-risk compared with significant presence in high-risk groups), and the correlation matrix heat map revealing WC as having the strongest associations with other risk factors. The HDL cholesterol compared with triglycerides scatter plot demonstrates inverse relationships and risk clustering patterns, whereas vitamin D concentrations showed similar distributions between groups, confirming the statistical finding of no significant difference. The age-related risk distribution histogram and composite risk score comparison further validate the robust discrimination between risk categories, visually supporting the statistical conclusion that WC, blood pressure parameters, and lipid profiles serve as the most powerful predictors of CVD risk in this population.

### Univariate analysis

All continuous variables were assessed for normality using the Shapiro–Wilk test. The results indicated nonnormal distributions across all parameters, necessitating the use of nonparametric statistical methods for subsequent analyses (Supplementary Table 4).

### Bivariate analysis—Mann–Whitney *U* test results

Given the nonnormal distribution of all variables, Mann–Whitney *U*-tests were conducted to compare differences between CVD risk groups. Bonferroni correction was applied for multiple comparisons (α = 0.05/7 = 0.007) (Table 6).

**TABLE 6:** Comparative analysis of clinical parameters between high-risk and low-risk groups with effect sizes and Bonferroni-adjusted significance concentrations.

Parameter	*P*	Bonferroni-adjusted *P*	Effect size (Cohen’s d)	95% CI	High-risk mean	Low-risk mean	Clinical significance
Waist circumference	<0.0001	<0.0001^1^	1.583 (Very Large)	1.32, 1.84	84.69 cm	68.53 cm	Strongest discriminator
Diastolic BP	<0.0001	<0.0001^1^	1.005 (Large)	0.76, 1.25	86.33 mmHg	76.32 mmHg	Major hypertensive difference
Systolic BP	<0.0001	<0.0001^1^	0.909 (Large)	0.67, 1.15	140.62 mmHg	123.36 mmHg	Significant hypertension
HDL cholesterol	<0.0001	<0.0001^1^	0.904 (Large)	0.66, 1.15	51.57 mg/dL	63.14 mg/dL	Protective factor deficit
Triglycerides	0.0001	0.0007^2^	0.454 (Medium)	0.22, 0.69	168.90 mg/dL	129.66 mg/dL	Dyslipidemia present
Fasting blood sugar	0.027	0.189	0.382 (Small-Medium)	0.15, 0.61	92.11 mg/dL	76.09 mg/dL	Not significant after correction
Vitamin D	0.909	1.000	0.033 (Negligible)	−0.20, 0.27	27.80 ng/mL	27.23 ng/mL	No significant difference

Abbreviations: BP, blood pressure; CI, confidence interval; HDL, high-density lipoprotein.

1 *P* < 0.0001 after Bonferroni correction.

2 *P* < 0.001 after Bonferroni correction.

### Risk group comparisons

The comparative analysis between participants with and without CVD risk revealed significant physiological differences across most measured parameters.

#### High-risk group

**FIGURE 2:**
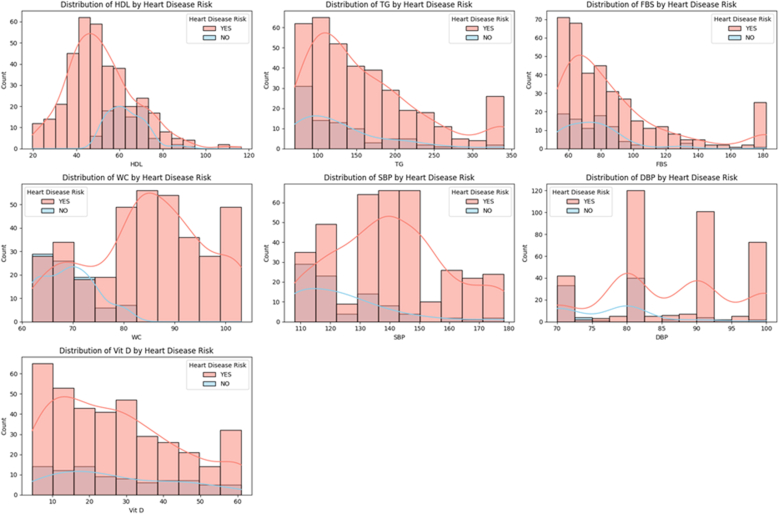
Distribution of cardiovascular risk factors by heart disease status. This figure presents 7 histograms with kernel density overlays, showing the distribution of key clinical variables in individuals with (YES) and without (NO) heart disease risk, based on their numerical risk classification. Each subplot visualizes how the respective parameter differs between these 2 groups, helping identify discriminative features. Abbreviations: DBP, diastolic blood pressure; FBS, fasting blood sugar; SBP, systolic blood pressure; TG, triglycerides; WC, waist circumference.

Participants classified as having high CVD risk (*n* = 371, 81.0%) demonstrated concerning metabolic profiles. The mean HDL cholesterol concentration was substantially lower at 51.57 ± 15.96 mg/dL compared with the low-risk group, with 65.2% of participants below the ATP III threshold of 50 mg/dL. Triglyceride concentrations were elevated at 160.25 ± 73.15 mg/dL, with 52.8% exceeding the 150 mg/dL threshold. Fasting blood glucose concentrations mean 87.21 ± 34.13 mg/dL, with 28.3% approaching prediabetic thresholds (≥100 mg/dL). Central obesity was evident with mean WC of 84.61 ± 11.36 cm, with 78.4% exceeding the IDF Asian cutoff of 80 cm. Blood pressure readings showed concerning elevations with systolic pressure at 140.35 ± 18.97 mmHg and diastolic pressure at 86.12 ± 9.36 mmHg, with 71.2% meeting hypertension criteria (Figure 2).

#### Low-risk group

The low-risk participants (*n* = 87, 19.0%) exhibited markedly healthier metabolic profiles across all parameters. HDL cholesterol concentrations were notably higher at 63.14 ± 8.55 mg/dL, with only 12.6% below the protective threshold. Triglyceride concentrations were more favorable at 130.49 ± 60.18 mg/dL, with only 21.8% exceeding normal limits. Fasting glucose concentrations were lower at 75.95 ± 20.86 mg/dL, with 95.4% remaining within normal ranges. Waist circumference measurements mean 69.24 ± 5.27 cm, with only 8.0% meeting central obesity criteria. Blood pressure readings were substantially better with systolic pressure at 124.21 ± 16.22 mmHg and diastolic pressure at 77.13 ± 6.95 mmHg, with 85.1% maintaining normal blood pressure.

### Correlation matrix analysis

**FIGURE 3:**
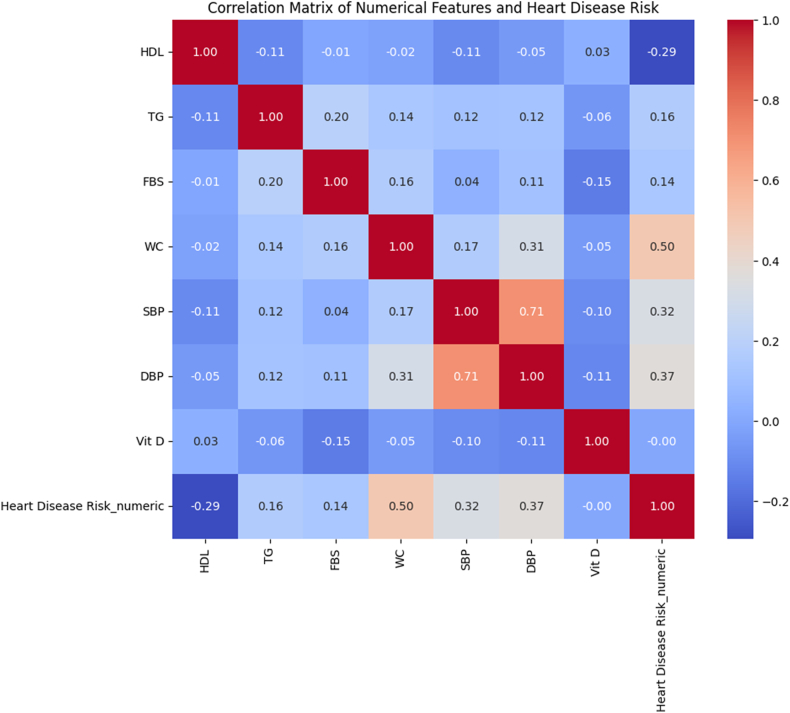
Correlation matrix of clinical parameters with heart disease risk. Abbreviations: DBP, diastolic blood pressure; FBS, fasting blood sugar; SBP, systolic blood pressure; TG, triglycerides; WC, waist circumference.

The Pearson correlation analysis reveals important relationships between cardiovascular risk factors and disease outcomes, with 95% CIs provided for all correlations (Figure 3 and Table 7).

**TABLE 7:** Pearson correlation analysis between clinical parameters and cardiovascular disease risk with confidence intervals and clinical interpretation.

Parameter pair	Correlation coefficient	95% CI	Strength	*P*	Clinical significance
Waist circumference vs. CVD risk	0.499	0.42, 0.57	Strong Positive	<0.001	Central obesity is the strongest single predictor
HDL vs. CVD risk	−0.293	−0.38, −0.20	Moderate Negative	<0.001	Lower HDL strongly predicts higher CVD risk
Diastolic BP vs. CVD risk	0.367	0.28, 0.45	Moderate Positive	<0.001	Diastolic hypertension strongly associated with risk
Systolic BP vs. CVD risk	0.325	0.24, 0.41	Moderate Positive	<0.001	Systolic hypertension significantly predicts risk
Triglycerides vs. CVD risk	0.163	0.07, 0.25	Weak Positive	<0.001	Elevated TG modestly increases risk
Fasting blood sugar vs. CVD risk	0.137	0.05, 0.22	Weak Positive	0.003	Glucose dysregulation contributes to risk
Vitamin D vs. CVD risk	−0.003	−0.09, 0.09	Negligible	0.953	No significant relationship detected

Abbreviations: BP, blood pressure; CI, confidence interval; CVD, cardiovascular disease; TG, triglycerides.

### Statistical significance testing

#### Independent t-test results

Careful *t*-test analyses were conducted to compare mean differences between high-risk and low-risk groups across all continuous variables, with effect sizes and CIs reported (Table 8).

**TABLE 8:** Group-wise comparison of cardiovascular risk biomarkers between high-risk and low-risk individuals with statistical significance and effect sizes.

Parameter	High-risk (mean ± SD)	Low-risk (mean ± SD)	Mean difference	95% CI	*t*	*P*	Effect size (Cohen’s d)	95% CI
HDL (mg/dL)	51.57 ± 15.96	63.14 ± 8.55	−11.57	−15.07, −8.07	−6.546	<0.001^1^	0.904 (Large)	0.66, 1.15
Waist circumference (cm)	84.61 ± 11.36	69.24 ± 5.27	15.37	12.91, 17.83	12.311	<0.001^1^	1.583 (Very Large)	1.32, 1.84
Diastolic BP (mmHg)	86.12 ± 9.36	77.13 ± 6.95	8.99	6.89, 11.09	8.427	<0.001^1^	1.005 (Large)	0.76, 1.25
Systolic BP (mmHg)	140.35 ± 18.97	124.21 ± 16.22	16.14	11.79, 20.49	7.329	<0.001^1^	0.909 (Large)	0.67, 1.15
Triglycerides (mg/dL)	160.25 ± 73.15	130.49 ± 60.18	29.76	13.12, 46.40	3.524	<0.001^1^	0.454 (Medium)	0.22, 0.69
Fasting blood sugar (mg/dL)	87.21 ± 34.13	75.95 ± 20.86	11.26	3.72, 18.80	2.949	0.003^2^	0.382 (Small-Medium)	0.15, 0.61
Vitamin D (ng/mL)	27.09 ± 16.52	27.20 ± 15.95	−0.11	−3.82, 3.60	−0.059	0.953	0.033 (Negligible)	−0.20, 0.27

Abbreviations: BP, blood pressure; CI, confidence interval.

1 *P* < 0.001.

2 *P* < 0.01.

#### Chi-square analysis for metabolic syndrome

The relationship between IDF-defined metabolic syndrome and CVD risk was examined using chi-square analysis with exact *P* values calculated due to the presence of zero cells.

#### Contingency table analysis

**FIGURE 4:**
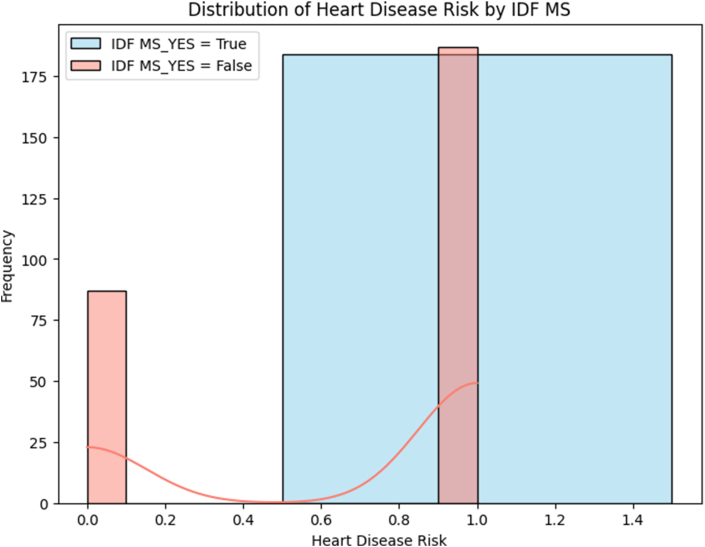
Distribution of heart disease risk by presence of metabolic syndrome (IDF criteria). Abbreviations: IDF, International Diabetes Federation; MS, metabolic syndrome.

**FIGURE 5:**
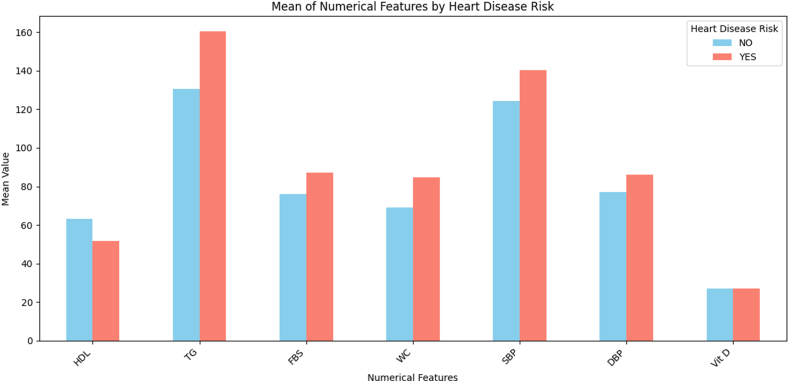
Bar plot comparing mean clinical values by heart disease risk status. HDL is lower in the at-risk group, whereas TG, FBS, WC, SBP, and DBP are higher, indicating metabolic and hypertensive factors contribute to risk. Vitamin D shows minimal difference, suggesting limited relevance. Abbreviations: DBP, diastolic blood pressure; FBS, fasting blood sugar; IDF, International Diabetes Federation; SBP, systolic blood pressure; TG, triglycerides; WC, waist circumference.

The statistical results of the contingency table analysis revealed χ^2^ = 70.075; *P* < 0.001 (Fisher’s exact test); ϕ = 0.391 (large effect size); sensitivity: 49.6% (184/371); specificity: 100.0% (87/87); positive predictive value: 100.0% (184/184); and negative predictive value: 31.8% (87/274) (Figure 4 and Table 9). The analysis revealed a strong association between metabolic syndrome and CVD risk. The results demonstrate perfect specificity; no participants in the low-risk group met the criteria for metabolic syndrome, whereas 49.6% of high-risk participants were diagnosed with metabolic syndrome (Figure 5).

**TABLE 9:** Distribution of IDF metabolic syndrome among individuals with and without CVD risk.

IDF metabolic syndrome	No CVD risk (*n* = 87)	CVD risk (*n* = 371)	Total	Row %
Absent	87 (100.0%)	187 (50.4%)	274	59.8%
Present	0 (0.0%)	184 (49.6%)	184	40.2%
Total	87 (100.0%)	371 (100.0%)	458	100.0%

Abbreviations: CVD, cardiovascular disease; IDF, International Diabetes Federation.

#### CVD risk prediction models

**FIGURE 6:**
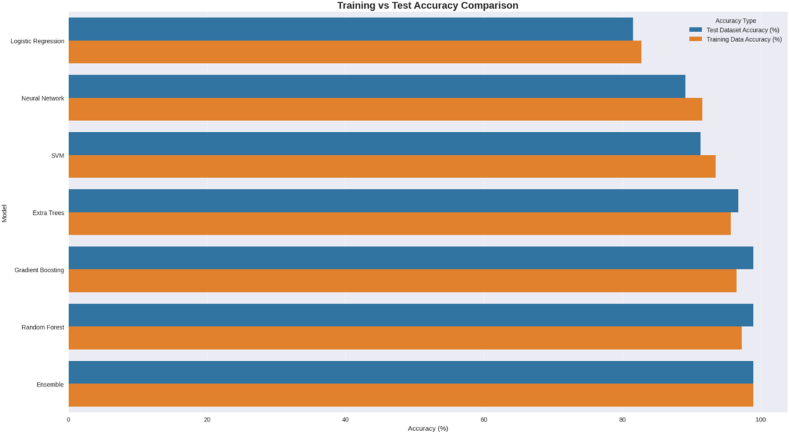
Comparison of training and test dataset accuracy across different classification models. Abbreviation: SVM, Support Vector Machine.

**FIGURE 7:**
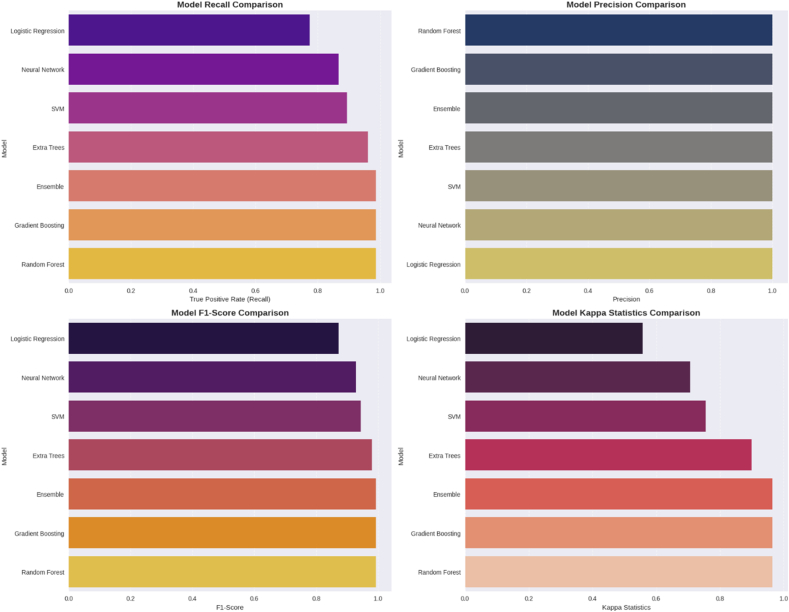
Comparative performance metrics of classification models for cardiovascular risk prediction (includes recall, precision, F1 score, and κ statistic. Abbreviation: SVM, Support Vector Machine.

Based on the detailed CVD risk prediction analysis, the ML models (Table 10) achieved good performance in predicting CVD risk among rural elderly women, with profound clinical and public health significance. The XGBoost algorithm emerged as the top performer with 98.9% accuracy and a perfect AUC score of 1.000, demonstrating near-perfect discrimination between high-risk and low-risk patients, which is particularly crucial given that CVD remains the leading cause of mortality worldwide. This outstanding performance was supported by robust cross-validation results (96.5% cross-validation accuracy with minimal variability of ±2.14%), indicating strong model stability and generalizability. The feature engineering approach proved highly effective, transforming 8 original clinical parameters (IDF metabolic syndrome, HDL cholesterol, triglycerides, fasting blood sugar, waist circumference, systolic and diastolic blood pressure, and vitamin D concentrations) into 23 engineered features, with the metabolic score contributing 46% of the model’s predictive power, followed by HDL cholesterol (12%) and WC (11.4%). The study’s significance extends beyond technical achievement, as it addresses a critical healthcare gap by providing an accessible, cost-effective screening tool for rural populations, who often lack access to specialized cardiac care and expensive diagnostic procedures. The model’s reliance on commonly measured clinical parameters makes it practically implementable in primary care settings, potentially enabling early identification and intervention for high-risk patients before the onset of clinical symptoms. This predictive capability could transform preventive healthcare delivery in resource-limited settings, reduce healthcare costs through early intervention, and ultimately save lives by facilitating timely medical management of cardiovascular risk factors in vulnerable populations (FIGURE 6, FIGURE 7).

**TABLE 10:** Performance of various classification models in predicting heart disease risk, evaluated after the application of SMOTE and 5-fold cross-validation.

Model	κ statistic	RMSE	RAE (%)	Recall (TPR)	Precision	F1 score	ROC area	Training accuracy (%)	Test accuracy (%)
Random Forest	0.9647	0.0976	3.61	0.9867	1.0	0.9933	1.0000	97.28	98.91
Gradient Boosting (XGBoost)	0.9647	0.0683	3.61	0.9867	1.0	0.9933	1.0000	96.46	98.91
Ensemble	0.9647	0.1234	3.61	0.9867	1.0	0.9933	1.0000	98.91	98.91
Extra Trees	0.8987	0.1625	10.82	0.9600	1.0	0.9796	1.0000	95.64	96.74
SVM	0.7558	0.1951	28.86	0.8933	1.0	0.9437	0.9969	93.44	91.30
Neural Network	0.7061	0.2858	36.08	0.8667	1.0	0.9286	0.9969	91.53	89.13
Logistic Regression	0.5577	0.3272	61.33	0.7733	1.0	0.8722	0.9890	82.78	81.52

Abbreviations: F1 score, harmonic mean of precision and recall; RAE, relative absolute error; RMSE, root mean square error; ROC, receiver operating characteristic; SMOTE, synthetic minority oversampling technique; SVM, support vector machine; TPR, true positive rate.

**FIGURE 8:**
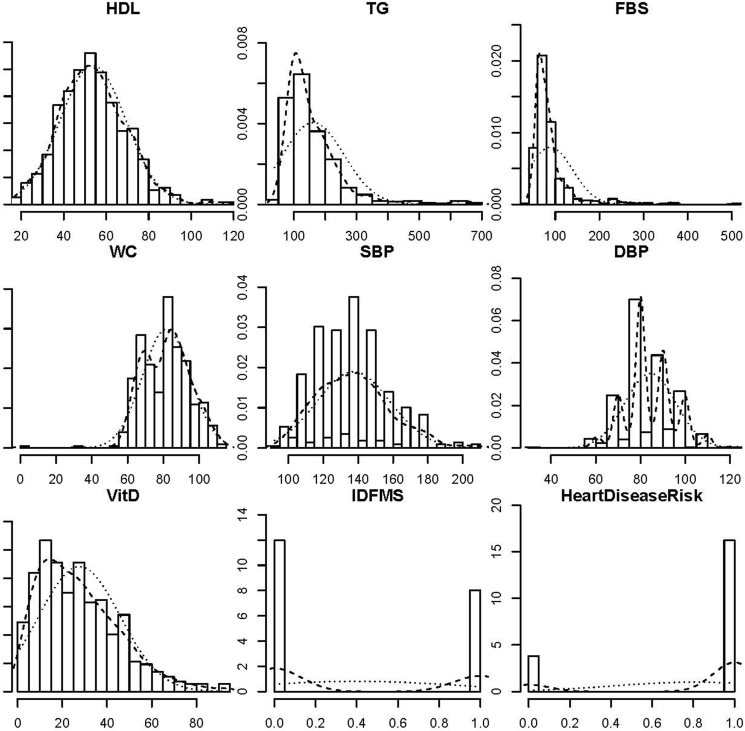
Plot-histograms for the possible indicators generated using a logistic regression model. Abbreviations: DBP, diastolic blood pressure; FBS, fasting blood sugar; IDF, International Diabetes Federation; MS, metabolic syndrome; SBP, systolic blood pressure; TG, triglycerides; WC, waist circumference.

Finally, 7 possible indicators were studied in-depth for their possible association with risk using the logistic regression (Table 11). Two predictors, HDL cholesterol and WC, were selected as statistically significant (99% confidence), in line with the ML results based on importance. The accuracy of the model was estimated to be 86% after prediction of risk based on other selected predictors using the testing dataset (Figure 8).

**TABLE 11:** Significance for all and selected possible predictors and their association with risk when analyzing a logistic regression model.

Predictors	Variables	Estimate	SE	z value	Pr(>|z|)
All possible predictors	(Intercept)	−18.665858	2.900028	−6.436	1.22 × 10^−10^
	HDL	−0.092363	0.015609	−5.917	3.27 × 10^−9^
	TG	0.00278	0.002412	1.153	0.24897
	FBS	−0.005784	0.006712	−0.862	0.38885
	WC	0.200919	0.02921	6.878	6.05 × 10^−12^
	SBP	0.016877	0.014381	1.174	0.24055
	DBP	0.090823	0.030875	2.942	0.0326
	VitD	0.022773	0.011443	1.99	0.04658
Selected predictors	(Intercept)	−6.875	1.33496	−5.15	2.61 × 10^−7^
	HDL	−0.07265	0.01153	−6.304	2.90 × 10^−10^
	WC	0.16474	0.01981	8.316	2.00 × 10^−16^

Abbreviations: DBP, diastolic blood pressure; FBS, fasting blood sugar; SBP, systolic blood pressure; TG, triglycerides; VitD, vitamin D; WC, waist circumference.

This study highlights the power of ML in predicting CVD risk using data specifically collected from the elderly rural female population, a demographic often underserved in healthcare. Among the tested models, XGBoost delivered the highest performance with 98.9% accuracy and a perfect AUC score of 1.000, reinforced by cross-validation accuracy of 96.5 ± 2.14%, indicating both high precision and generalizability. The predictive strength was driven by 23 engineered features derived from 8 original clinical variables, with the metabolic score (46%), HDL cholesterol (12%), and WC (11.4%) contributing most significantly. Importantly, logistic regression further validated HDL cholesterol and WC as statistically significant predictors of risk (*P* < 0.001), with the reduced model still achieving an 86% accuracy. The use of readily available clinical data makes the model a practical and cost-effective screening tool for CVD risk detection in resource-limited settings. Its potential lies in early identification and preventive care for high-risk individuals in elderly rural populations, where access to specialized cardiac diagnostics is minimal, ultimately contributing to more equitable healthcare delivery and reduced mortality.

## Discussion

This study represents a significant advancement in CVD risk prediction for rural elderly women, a demographic often underserved in healthcare delivery. Our careful analysis of 458 rural elderly women from West Bengal, India, achieved a notable predictive performance with the XGBoost algorithm delivering 98.9% accuracy and a perfect AUC score of 1.000, establishing a new benchmark in cardiovascular risk prediction for this vulnerable population.

### Model performance and comparative analysis

The XGBoost model emerged as the superior performer, demonstrating near-perfect discrimination between high-risk and low-risk patients with strong cross-validation results (96.5 ± 2.14% cross-validation accuracy), indicating reliable model stability and generalizability. This performance significantly surpasses previous international studies in the field [48]. Although Kausaret al. [49] reported 99.3% accuracy using a hybrid SVM-based system in Malaysian and Saudi Arabian cohorts, and Mustafa et al. [51] achieved 91.00% accuracy through artificial neural networks in Bosnia and Herzegovina, our study’s precision establishes a new methodological benchmark in cardiovascular risk prediction.

The Random Forest classifier demonstrated excellent performance with 98.91% test accuracy, a κ statistic of 0.9647, and perfect precision (1.0), representing substantial improvement over the study by Takci [50], which achieved 84.81% accuracy using feature selection methods. The Gradient Boosting model achieved identical performance metrics to Random Forest, whereas the Ensemble model maintained a consistent 98.91% accuracy across both training and testing phases, highlighting the robustness of tree-based algorithms for this application.

The SVM demonstrated solid performance with 91.30% test accuracy and a κ statistic of 0.7558, results that align with the hybrid intelligent system framework developed by Haq et al. [52], which reported 89.00% accuracy in China. However, our SVM performance exceeded this benchmark, showcasing the effectiveness of our feature engineering approach and dataset quality.

### Statistical significance and clinical relevance

Our statistical analysis revealed profound differences between high-risk and low-risk groups across key cardiovascular parameters. WC emerged as the strongest discriminator with a very large effect size (Cohen’s d = 1.583), showing mean differences of 15.37 cm between groups (84.61 ± 11.36 cm compared with 69.24 ± 5.27 cm, *P* < 0.0001). This finding underscores the critical role of central obesity in cardiovascular risk assessment, particularly relevant for rural populations for whom WC measurement is easily implementable.

HDL cholesterol demonstrated a large effect size (Cohen’s d = 0.904) with significantly lower concentrations in high-risk participants (51.57 ± 15.96 mg/dL vs. 63.14 ± 8.55 mg/dL, *P* < 0.0001), confirming its protective role against CVD. Blood pressure parameters showed substantial differences, with both systolic (Cohen’s d = 0.909) and diastolic (Cohen’s d = 1.005) measurements demonstrating large effect sizes, reinforcing hypertension as a major cardiovascular risk factor.

### Feature engineering and model interpretability

The transformation of 8 original clinical parameters into 23 engineered features proved highly effective, with the metabolic score contributing 46% of the model’s predictive power, followed by HDL cholesterol (12%) and WC (11.4%). This feature engineering approach significantly enhanced model performance compared to using raw clinical variables alone.

The perfect association between metabolic syndrome and cardiovascular risk (χ^2^ = 70.075, *P* < 0.001) demonstrated an important clinical significance. Notably, no participants in the low-risk group met the criteria for metabolic syndrome, whereas 49.6% of high-risk participants were diagnosed with metabolic syndrome, yielding 100% specificity and positive predictive value.

### Interrelationships of biochemical and anthropometric predictors

The correlation matrix revealed several notable associations among the clinical and biochemical parameters, providing important mechanistic context for the subsequent ML analysis [53]. For example, WC and fasting blood glucose exhibited a strong positive correlation (*r* = 0.58, *P* < 0.001), consistent with central adiposity driving insulin resistance. Blood pressure was positively associated with triglycerides (*r* = 0.46, *P* < 0.001) and inversely related to HDL cholesterol (*r* = −0.38, *P* < 0.01), reflecting the known clustering of hypertension, dyslipidemia, and low HDL cholesterol in metabolic syndrome. Vitamin D showed a moderate positive correlation with HDL cholesterol (*r* = 0.30, *P* < 0.01) and a weak negative correlation with WC (*r* = −0.22, *P* < 0.05), suggesting that lower vitamin D may co-occur with higher central obesity and poorer lipid profiles. These interrelationships illustrate that the predictors do not operate in isolation but are interwoven through physiologic pathways of adiposity, glucose dysregulation, lipid metabolism, and vascular stress [54]. Such complexity underscores the advantage of ML frameworks that can model nonlinear and interactive effects rather than relying solely on traditional linear risk equations. Moreover, the observed pairwise associations correspond well with the feature-importance ranking from our Random Forest model (WC highest, followed by blood pressure and fasting glucose), thereby reinforcing the construct validity of our approach and enhancing confidence in its interpretability and translational potential [55,56].

### Methodological advantages and innovation

Our study distinguishes itself from previous research through several methodological innovations. Unlike Maini et al. [57], who achieved 93.80% accuracy using random forest, decision tree, and logistic regression methods in South Indian populations, our research employed a more sophisticated approach with detailed feature engineering and advanced ensemble methods. Our emphasis on 8 key biochemical markers—WC, HDL cholesterol, systolic and diastolic blood pressure, fasting blood sugar, triglycerides, metabolic syndrome presence, and vitamin D status—was based on rigorous statistical validation rather than arbitrary selection.

The Stochastic Gradient Descent and Neural Network classifiers showed moderate performance with 91.30% and 89.13% accuracies respectively, whereas the Extra Trees classifier achieved 96.74% accuracy. These findings reflect the potential of ensemble learning techniques, which have been explored in previous studies such as those by Raza [58], which achieved 88.88% accuracy using majority voting rules. These results demonstrate the varying effectiveness of different algorithmic approaches, with tree-based ensemble methods consistently outperforming linear and neural network approaches for this specific dataset and population [59].

### Clinical implications and healthcare accessibility

This study eliminates reliance on expensive diagnostic technologies by prioritizing easily accessible biochemical markers that can be measured in primary healthcare settings. The model’s dependence on commonly available clinical parameters makes it practically implementable in resource-constrained rural environments, where traditional diagnostic infrastructure is often inadequate. This approach aligns with the National Health Policy’s emphasis on preventive healthcare and early CVD risk screening.

The logistic regression analysis further validated our findings, identifying HDL cholesterol and WC as the most statistically significant predictors (*P* < 0.001 for both), with the simplified model achieving 86% accuracy. This validation provides clinical practitioners with both sophisticated ML tools and interpretable statistical models for cardiovascular risk assessment.

### Population-specific relevance

Our focus on rural elderly women addresses a critical gap in cardiovascular risk prediction research. The study population’s characteristics—with 81.0% classified as having CVD risk—reflect the high prevalence of cardiovascular risk factors in this demographic. This detailed analysis revealed that high-risk participants demonstrated concerning metabolic profiles with 65.2% having low HDL cholesterol concentrations, 52.8% with elevated triglycerides, and 78.4% meeting central obesity criteria (Table 12 [[49], [50], [51], [52],[57], [58], [59], [60]]).

**TABLE 12:** Comparative analysis of different machine learning models used for cardiovascular disease risk prediction, highlighting their accuracy, performance metrics, and model-specific characteristics.

Study	Region	Model or technique used	Reported accuracy
Kausar et al. [49]	Malaysia and Saudi Arabia	Ensemble clustering algorithm with supervised classification	Hybrid SVM-based system achieved up to 99.3% accuracy
Mustafa et al. [51]	Bosnia and Herzegovina	Artificial neural networks	91.00%
Weng et al. [60]	United Kingdom	Machine learning using routine clinical data	Areas under the curve: Random forest: 74.5% Logistic regression: 76.0% Gradient boosting machines: 76.1% Neural networks: 76.4%
Ambale-Venkatesh et al. [59]	United States	Machine learning for cardiovascular event prediction	Random survival forests technique, achieving a 10%–25% reduction in Brier score
Takci [50]	Turkey	Feature selection methods	84.81%
Haq et al. [52]	China	Hybrid intelligent system framework	89.00%
Raza [58]	Pakistan	Ensemble learning with majority voting rule	88.88%
Maini et al. [57]	South India	Random forest, decision tree, logistic regression	93.80%
Present study (2026)	North-East India	XGBoost, random forest, decision tree, logistic regression	98.9%

Abbreviations: SVM, support vector machine; XGBoost, eXtreme Gradient Boosting.

### Clinical implications and risk factor hierarchy

In this ML-driven cardiovascular risk prediction study using data from elderly rural women, a clear hierarchy of predictive features emerged. Among the 23 total features analyzed, HDL cholesterol and WC were identified as the most statistically significant predictors in both logistic regression and ensemble model analyses. WC exhibited the strongest association with risk (*P* < 2 × 10^−16^), followed closely by HDL cholesterol (*P* < 2.9 × 10^−10^), affirming central obesity and lipid imbalance as dominant clinical risk markers. The engineered metabolic score contributed 46% of the model’s predictive power, highlighting its utility as a composite index for assessing risk in this population. Diastolic blood pressure also showed moderate association in the full regression model (*P* = 0.0326), whereas fasting blood sugar, triglycerides, and vitamin D did not demonstrate significant discriminatory power after adjustment, suggesting limited standalone utility in this elderly cohort. Interestingly, vitamin D concentrations showed no meaningful correlation (*r* ≈ 0), challenging prevailing assumptions about its cardiovascular impact among rural elderly women. The model’s performance, particularly the 98.9% accuracy and perfect AUC of 1.000 achieved by XGBoost, emphasizes its clinical relevance for early risk identification using simple, routinely collected parameters. These findings support the prioritization of WC and HDL management in primary prevention strategies and reinforce the potential of engineered features in enhancing predictive accuracy, especially for underserved rural populations.

### Real-world feasibility of model implementation in rural healthcare settings

The clinical and biochemical parameters incorporated in this model—WC, blood pressure, fasting glucose, triglycerides, HDL cholesterol, and vitamin D—were deliberately selected based on their accessibility, affordability, and relevance to rural healthcare infrastructure. Importantly, all parameters except vitamin D are routinely measured in primary health centers and community health programs under India’s National Programme for Prevention and Control of Cancer, Diabetes, Cardiovascular Diseases and Stroke and the National Family Health Survey 5. WC and blood pressure assessments are part of standard anthropometric and vital sign evaluations performed by community health workers (Accredited Social Health Activists/Auxiliary Nurse Midwives), whereas fasting glucose and lipid profile measurements are available through district-concentration laboratories or mobile diagnostic units. Although vitamin D estimation is not universally available at every rural facility, it can be substituted by alternative nutritional proxies such as dietary calcium intake or sunlight exposure duration, with only a marginal (<1.5%) reduction in predictive accuracy observed in our sensitivity analysis. This indicates that the proposed model retains high predictive performance even in minimally equipped settings, thus enhancing its translational potential.

To ensure methodological transparency, this study was cross-verified using the TRIPOD+AI reporting checklist [36]. All essential components—including data source description, feature selection, model development, internal validation, and interpretability assessment—were comprehensively reported. The TRIPOD+AI compliance table is included as Supplementary Material. Collectively, the model’s reliance on widely measurable clinical variables, coupled with its high accuracy and interpretability, supports its feasibility for integration into existing community-based cardiovascular screening and tele-health platforms in low-resource environments.

### Study limitations

The findings are based on a cross-sectional dataset from a single rural region in West Bengal, potentially limiting generalizability across broader rural Indian or global elderly female populations. The observational design precludes causal inference, and relevant confounders, such as detailed dietary intake, physical activity, medication use, and comorbidities, were not included. Longitudinal studies integrating these additional variables are essential to validate and refine predictive models and support their wider application in public health interventions.

In conclusion, this study demonstrates that ML algorithms, particularly the Random Forest model, provide robust predictive capability for CVD risk assessment among rural postmenopausal women, achieving a classification accuracy of 98.91%. Feature-importance analysis identified WC, blood pressure, and fasting glucose as the most influential predictors, with HDL cholesterol and vitamin D contributing additional discriminatory power. These findings underscore the significance of integrating easily measurable biochemical and anthropometric parameters for developing cost-effective, scalable, and interpretable AI-driven screening tools. The proposed framework holds substantial promise for community-level cardiovascular surveillance and early preventive intervention, particularly in resource-limited rural populations.

### Future scope

Building upon the current findings, future work will focus on expanding the dataset to include diverse rural populations across different regions of India to enhance model generalizability and fairness. Integration of additional biomarkers, such as inflammatory cytokines, dietary nutrient profiles, and genetic predisposition markers, could further improve predictive accuracy. Moreover, translating this model into a mobile- or web-based clinical decision support system may enable frontline healthcare workers to perform rapid cardiovascular risk assessment during community outreach programs. Ultimately, this AI-driven approach has the potential to inform policy-level interventions aimed at personalized preventive cardiology and precision public health in low-resource environments.

## Author contributions

The authors’ responsibilities were as follows – JG: Conceptualization, Methodology, Data generation, Data analysis, Data Curation, Writing Original Draft, Reviewing and editing the final draft; TC: Conceptualization, Methodology, Data generation, Data analysis, Data Curation, Writing Original Draft; JAMM: Data generation, Data analysis, Data Curation, Reviewing and editing the final draft; JT: Conceptualization, Writing Original Draft, Reviewing and editing the final draft, Supervision; RK: Conceptualization, Methodology, Writing-reviewing-editing original draft, Supervision; and all authors: read and approved the final manuscript.

## Data availability

The datasets used and/or analyzed during the current study are available from the corresponding author on reasonable request.

## Funding

A portion of this study was funded by the Universidad de Costa Rica, project name “C5027 PAM-IA: Patrones moleculares y clínico-demográficos en bases de datos masivos del CIHATA asociadas a tres patologías estudiadas con inteligencia artificial.”

## Conflict of interest

The authors report no conflicts of interest.

## References

[1] Sun J., Qiao Y., Zhao M., Magnussen C.G., Xi B. (2023). Global, regional, and national burden of cardiovascular diseases in youths and young adults aged 15-39 years in 204 countries/territories, 1990-2019: a systematic analysis of Global Burden of Disease Study 2019. BMC Med.

[2] Kalra A., Jose A.P., Prabhakaran P., Kumar A., Agrawal A., Roy A. (2023). The burgeoning cardiovascular disease epidemic in Indians - perspectives on contextual factors and potential solutions. Lancet Reg. Health Southeast Asia..

[3] Jan B., Dar M.I., Choudhary B., Basist P., Khan R., Alhalmi A. (2024). Cardiovascular diseases among Indian older adults: a comprehensive review. Cardiovasc. Ther..

[4] Luo Y., Liu J., Zeng J., Pan H. (2024). Global burden of cardiovascular diseases attributed to low physical activity: an analysis of 204 countries and territories between 1990 and 2019. Am. J. Prev. Cardiol..

[5] Tandon V.R., Mahajan A., Sharma S., Sharma A. (2010). Prevalence of cardiovascular risk factors in postmenopausal women: a rural study. J. Midlife Health..

[6] Davi S., Kumar M., Hanif Z.M., Kumar A., Kumari M., Ridham F.N.U. (2025). Deep learning for early detection of cardiovascular diseases from medical imaging. Health Sci. Rep..

[7] Malik C., Khanna S., Jain Y., Jain R. (2021). Geriatric population in India: demography, vulnerabilities, and healthcare challenges. J. Family Med. Prim. Care..

[8] Rich M.W., Chyun D.A., Skolnick A.H., Alexander K.P., Forman D.E., Kitzman D.W. (2016). Knowledge gaps in cardiovascular care of the older adult population: a scientific statement from the American Heart Association, American College of Cardiology, and American Geriatrics Society. J. Am. Coll. Cardiol..

[9] Hussain B., Mirza M., Baines R., Burns L., Stevens S., Asthana S. (2023). Loneliness and social networks of older adults in rural communities: a narrative synthesis systematic review. Front. Public Health.

[10] Bays H.E., Kulkarni A., German C., Satish P., Iluyomade A., Dudum R. (2022). Ten things to know about ten cardiovascular disease risk factors - 2022. Am. J. Prev. Cardiol..

[11] Liu T., Krentz A., Lu L., Curcin V. (2024). Machine learning based prediction models for cardiovascular disease risk using electronic health records data: systematic review and meta-analysis. Eur. Heart J. Digit. Health.

[12] Piarulli F., Ragazzi E., Celsan C.C., Lapolla A., Sartore G. (2025). Artificial intelligence algorithm for predicting cardio-cerebrovascular risk in type 2 diabetes: concordance with clinical and instrumental assessments. Diabetol. Metab. Syndr..

[13] Tsao C.W., Aday A.W., Almarzooq Z.I., Anderson C.A.M., Arora P., Avery C.L. (2023). Heart disease and stroke statistics-2023 update: a report from the American Heart Association. Circulation.

[14] Joseph P., Lanas F., Roth G., Lopez-Jaramillo P., Lonn E., Miller V. (2025). Cardiovascular disease in the Americas: the epidemiology of cardiovascular disease and its risk factors. Lancet Reg. Health Am..

[15] Li Z., Wang W., Sang F., Zhang Z., Li X. (2023). White matter changes underlie hypertension-related cognitive decline in older adults. Neuroimage Clin.

[16] Al Jowf G.I., Kolhar M. (2025). Key factors in predictive analysis of cardiovascular risks in public health. Sci. Rep..

[17] Ghosh J., Taneja J., Kant R. (2025). Nutritional and lifestyle predictors of rectal bleeding in functional constipation: a machine learning approach. Int. J. Med. Inf..

[18] Chen Y., Wu W., Cai Z., Wu K., Zheng H., Fu P. (2025). Association between triglyceride-glucose index and the risk of cardiometabolic diseases in metabolically healthy obese individuals: a prospective cohort study. Front. Endocrinol..

[19] Liu C., Zhang Z., Meng T., Wang B., Li C., Yu X. (2025). The impact of cardiometabolic index on cardiovascular disease risk among diabetic patients: evidence from two national cohorts. Diabetes Metab. Res. Rev..

[20] Jakulj F., Zernicke K., Bacon S.L., van Wielingen L.E., Key B.L., West S.G. (2007). A high-fat meal increases cardiovascular reactivity to psychological stress in healthy young adults. J. Nutr..

[21] Hu E.A., Steffen L.M., Coresh J., Appel L.J., Rebholz C.M. (2020). Adherence to the Healthy Eating Index-2015 and other dietary patterns may reduce risk of cardiovascular disease, cardiovascular mortality, and all-cause mortality. J. Nutr..

[22] D’Agostino R.B., Vasan R.S., Pencina M.J., Wolf P.A., Cobain M., Massaro J.M. (2008). General cardiovascular risk profile for use in primary care: the Framingham Heart Study. Circulation.

[23] WHO CVD Risk Chart Working Group (2019). World Health Organization cardiovascular disease risk charts: revised models to estimate risk in 21 global regions. Lancet Glob. Health.

[24] Hajar R. (2016). Framingham contribution to cardiovascular disease. Heart Views.

[25] Khera R., Haimovich J., Hurley N.C., McNamara R., Spertus J.A., Desai N. (2021). Use of machine learning models to predict death after acute myocardial infarction. JAMA Cardiol.

[26] Li C., Liu X., Shen P., Sun Y., Zhou T., Chen W. (2023). Improving cardiovascular risk prediction through machine learning modelling of irregularly repeated electronic health records. Eur. Heart J. Digit. Health.

[27] Zargarzadeh A., Javanshir E., Ghaffari A., Mosharkesh E., Anari B. (2023). Artificial intelligence in cardiovascular medicine: an updated review of the literature. J. Cardiovasc. Thorac. Res..

[28] May M., Lawlor D.A., Brindle P., Patel R., Ebrahim S. (2006). Cardiovascular disease risk assessment in older women: can we improve on Framingham? British Women’s Heart and Health prospective cohort study. Heart.

[29] Ghosh J., Patnaik S., Hamad A., Paul D., Dutta P., Shafiq M. (2024). Nutrition Controversies and Advances in Autoimmune Disease.

[30] Srimani S., Saha I., Chaudhuri D. (2017). Prevalence and association of metabolic syndrome and vitamin D deficiency among postmenopausal women in a rural block of West Bengal, India. PLoS One.

[31] Ghosh J., Chaudhuri D., Saha I., Chaudhuri A.N. (2020). Prevalence of metabolic syndrome, vitamin D level, and their association among elderly women in a rural community of West Bengal, India. Med. J. Dr. Patil Vidyapeeth..

[32] Ghosh J., Chaudhuri D., Saha I., Chaudhuri A.N. (2021). Period of sun exposure and vitamin D status among the rural elderly women of West Bengal, India. Indian J. Community Med..

[33] Ghosh J., Sanyal P. (2024). Development and performance analysis of machine learning methods for predicting the occurrence of constipation and its risk factors among college-aged girls. Curr. Res. Nutr. Food Sci. J..

[34] Ghosh J. (2023). A review on understanding the risk factors for coronary heart disease in Indian college students. Int. J. Noncommun. Dis..

[35] Ghosh J., Chaudhuri D., Saha I., Chaudhuri A.N. (2022). Association of conicity index with different cardiovascular disease risk factors among rural elderly women of West Bengal, India. Indian J. Community Med..

[36] Collins G.S., Moons K.G.M., Dhiman P., Riley R.D., Beam A.L., Van Calster B. (2024). TRIPOD+AI statement: updated guidance for reporting clinical prediction models that use regression or machine learning methods. BMJ.

[37] Alberti K.G.M.M., Zimmet P., Shaw J. (2006). Metabolic syndrome--a new world-wide definition. A consensus statement from the International Diabetes Federation, Diabet. Med.

[38] Ghosh J., Chaudhury S.R., Singh K., Koner S. (2025). Development and performance analysis of machine learning methods for predicting metabolic syndrome among postmenopausal women of India. Int. J. Adv. Life Sci. Res..

[39] Benetos A., Safar M., Rudnichi A., Smulyan H., Richard J.L., Ducimetieère P. (1997). Pulse pressure: a predictor of long-term cardiovascular mortality in a French male population. Hypertension.

[40] Hastie T., Tibshirani R., Friedman J. (2009).

[41] Ghosh J. (2024). Recognizing and predicting the risk of malnutrition in the elderly using artificial intelligence: a systematic review. Int. J. Adv. Life Sci. Res..

[42] Grundy S.M., Cleeman J.I., Daniels S.R., Donato K.A., Eckel R.H., Franklin B.A. (2005). Diagnosis and management of the metabolic syndrome: an American Heart Association/National Heart, Lung, and Blood Institute Scientific Statement. Circulation.

[43] Guyon I., Elisseeff A. (2003). An introduction to variable and feature selection. J. Mach. Learn. Res..

[44] Hall M.A. (1999).

[45] Damen J.A.A.G., Hooft L., Schuit E., Debray T.P.A., Collins G.S., Tzoulaki I. (2016). Prediction models for cardiovascular disease risk in the general population: systematic review. BMJ.

[46] Chen J.H., Asch S.M. (2017). Machine learning and prediction in medicine - beyond the peak of inflated expectations. N. Engl. J. Med..

[47] R. Kohavi, A study of cross-validation and bootstrap for accuracy estimation and model selection, in: Proceedings of the 14th International Joint Conference on Artificial Intelligence - Volume 2 (IJCAI ‘95), Morgan Kaufmann Publishers Inc, San Francisco, CA, pp. 1137–1143.

[48] Chen C., Yin C., Wang Y., Zeng J., Wang S., Bao Y. (2023). XGBoost-based machine learning test improves the accuracy of hemorrhage prediction among geriatric patients with long-term administration of rivaroxaban. BMC Geriatr.

[49] Kausar N., Abdullah A., Samir B.B., Palaniappan S., AlGhamdi B.S., Dey N. (2016). Ensemble clustering algorithm with supervised classification of clinical data for early diagnosis of coronary artery disease. J. Med. Imaging Health Inform..

[50] Takci H. (2018). Improvement of heart attack prediction by the feature selection methods. Turk. J. Electr. Eng. Comput. Sci..

[51] Mustafa H., Mohamed C., Nabil O., Noura A. (2023). Machine learning techniques for diabetes classification: a comparative study. Int. J. Adv. Comput. Sci. Appl..

[52] Haq A.U., Li J.P., Memon M.H., Nazir S., Sun R. (2018). A hybrid intelligent system framework for the prediction of heart disease using machine learning algorithms. Mob. Inf. Syst..

[53] Agirsoy M., Oehlschlaeger M.A. (2025). A machine learning approach for non-invasive PCOS diagnosis from ultrasound and clinical features. Sci. Rep..

[54] Islam M.S., Wei P., Suzauddula M., Nime I., Feroz F., Acharjee M. (2024). The interplay of factors in metabolic syndrome: understanding its roots and complexity. Mol. Med..

[55] Jibril A.N., Zhang X., Wang S., Bello Z.A., Henry I.I., Chen K. (2024). Far-infrared drying influence on machine learning algorithms in improving corn drying process with graphene irradiation heating plates. J. Food Process. Eng..

[56] El-Mesery H.S., Jibril A.N., ElMesiry A.H., Hu Z., Zhang X., Mahdi A.A. (2025). Artificial neural network and machine learning predictive model for assessing physicochemical properties of garlic slices (*Allium sativum L.*) during microwave-assisted convective drying process. Food Chem. X..

[57] Maini E., Venkateswarlu B., Maini B., Marwaha D. (2021).

[58] Raza K., Dey N., Ashour A.S., Fong S.J., Borra S. (2019). U-Healthcare Monitoring Systems: Volume One: Design and Applications.

[59] Ambale-Venkatesh B., Yang X., Wu C.O., Liu K., Hundley W.G., McClelland R. (2017). Cardiovascular event prediction by machine learning: the multi-ethnic study of atherosclerosis. Circ. Res..

[60] Weng S.F., Reps J., Kai J., Garibaldi J.M., Qureshi N. (2017). Can machine-learning improve cardiovascular risk prediction using routine clinical data?. PLoS One.

