# Supplementary material for: Harnessing Clinical and Biochemical Data for Personalized Cardiovascular Risk Prediction: a Machine Learning Approach Toward Precision Nutrition
Source: J Nutr. 2026 Jan 13;156(3):101363. doi: 10.1016/j.tjnut.2026.101363 (PMC13014513; doi:10.1016/j.tjnut.2026.101363)
Supplement: Multimedia component 1 [file mmc1.docx]

**Harnessing Clinical and Biochemical Data for Personalized Cardiovascular Risk Prediction: A Machine Learning Approach Toward Precision Nutrition**

**Joyeta Ghosh^1^, Tinni Chaudhuri^2^, Jose Arturo Molina Mora^3^, JyotiTaneja^4 #^and Ravi Kant^5# ⁋^**

^1^Department of Dietetics and Applied Nutrition, Amity Institute of Applied Sciences (AIAS), Amity University - Kolkata Campus, Major Arterial Road, Action Area II, Kadampukur Village, Rajarhat, Newtown, Kolkata, West Bengal 700135, India.

^2^Dept. of Statistics, Amity Institute of Applied Sciences (AIAS), Amity University - Kolkata Campus, Major Arterial Road, Action Area II, Kadampukur Village, Rajarhat, Newtown, Kolkata, West Bengal 700135, India

^3^Facultad de Microbiología, Centro de Investigaciónen Enfermedades Tropicales (CIET) and Centro de investigaciónen Hematología y Trastornos Afines (CIHATA), Universidad de Costa Rica, San José, Costa Rica

^4^Department of Zoology, Daulat Ram College, University of Delhi, Delhi-110007 India

^5^Molecular Microbiology, School of Clinical and Experimental Sciences, Faculty of Medicine, University of Southampton, Southampton SO16 6YD, England

**^⁋^ Current Affiliation:** Faculty of Applied Sciences & Biotechnology, Shoolini University, Solan – 173229 Himachal Pradesh, India

**Correspondence: Ravi Kant;** [**R.kant.ac.uk@gmail.com**](mailto:R.kant.ac.uk@gmail.com)**;**

**Jyoti Taneja;**[**jyotitaneja@dr.du.ac.in**](mailto:jyotitaneja@dr.du.ac.in) **(# Equal CorrespondingAuthor)**

**Supplementary Table 1.** *Descriptive statistics of demographic and predictor variables in the training and test datasets used for machine learning model development. Continuous variables are presented as mean ± standard deviation (SD) and their corresponding p-values are shown in the last column.*

| **Parameter** | **Training Set (Mean ± SD)** | **Test Set**  **(Mean ± SD)** | **p-value** |
| --- | --- | --- | --- |
| **HDL** | 53.80±15.37 | 53.61±16.15 | 0.9145 |
| **TG** | 164.07±103.39 | 150.99±74.86 | 0.2547 |
| **FBS** | 89.64±52.65 | 86.77±37.90 | 0.6224 |
| **WC** | 80.97±13.16 | 84.19±13.86 | 0.0384 |
| **SBP** | 137.62±21.22 | 136.22±20.49 | 0.5678 |
| **DBP** | 84.53±10.97 | 84.00±12.81 | 0.6877 |
| **Vit D** | 28.05±18.40 | 26.27±16.83 | 0.3992 |
| **TG_to_HDL_Ratio** | 3.44±2.80 | 3.10±1.81 | 0.2648 |
| **Pulse_Pressure** | 53.09±15.31 | 52.22±16.76 | 0.6319 |

**Supplementary Table 2*:*** *Detailed Configuration of Seven Supervised Machine Learning Algorithms Used in the Study*

| **Model** | **Rationale** | **Hyperparameter Tuning Approach** | **Final Configuration** |
| --- | --- | --- | --- |
| **XGBoost** | Captures nonlinear feature interactions; robust to moderate class imbalance; provides gain-based feature importance. | Grid search + stratified 5-fold cross-validation; optimized for F1-score and ROC-AUC. | n_estimators = 200; learning_rate = 0.05; max_depth = 4; subsample = 0.8; colsample_bytree = 0.8; gamma = 0.1; min_child_weight = 3 |
| **Random Forest** | Strong ensemble baseline; low variance; interpretable; well-suited to clinical tabular data. | Grid search for n_estimators, max_features, min_samples_leaf using stratified 5-fold CV. | n_estimators = 200; max_features = ‘sqrt’; min_samples_leaf = 2; bootstrap = True; random_state = 42 |
| **Gradient Boosting** | Provides high predictive accuracy through additive weak learners. | Grid search over n_estimators, learning_rate, max_depth. | n_estimators = 200; learning_rate = 0.05; max_depth = 3 |
| **Support Vector Classifier (SVC)** | Effective for nonlinear classification using kernel methods. | Grid search across kernels (linear, polynomial, RBF, sigmoid) and regularization parameters (C, gamma). | kernel = ‘rbf’; C = 1.0; gamma = 0.1 |
| **Artificial Neural Network (ANN)** | Models complex nonlinear relationships; adaptable architecture. | Grid/random search on depth, neurons, dropout; early stopping used; 5-fold CV. | Architecture: 6–12–8–1; activation: ReLU (hidden) + sigmoid (output); optimizer = Adam (lr=0.001); epochs = 100; batch_size = 32; dropout = 0.2 |
| **K-Nearest Neighbours (KNN)** | Simple non-parametric classifier using neighborhood similarity. | Evaluated k ∈ {3,5,7,9} via stratified 5-fold CV; features standardized. | k = 5; metric = Euclidean; weights = uniform |
| **Decision Tree** | Fully interpretable baseline; identifies simple rule-based patterns. | Grid search for max_depth, min_samples_split, min_samples_leaf. | criterion = ‘gini’; max_depth = 6; min_samples_leaf = 5; random_state = 42 |

***Computational Environment and Reproducibility***

*All models were implemented using* ***Python 3.9.0*** *with libraries: scikit-learn v1.0.2, XGBoost v1.6.1, TensorFlow/Keras v2.6.0.
A fixed random seed (****random_state = 42****) ensured reproducibility for all stochastic algorithms.
Model tuning used* ***stratified 5-fold cross-validation****, and final results were evaluated on the fully independent test set.*

# Supplementary Table 3. TRIPOD-AI Compliance Summary for the Present Study

| **TRIPOD-AI Checklist Item** | **Description and Implementation in This Study** | **Manuscript Section** |
| --- | --- | --- |
| Title and Abstract | Title and abstract clearly identify the study as a machine learning–based cardiovascular disease (CVD) risk prediction model. The abstract summarizes objectives, methods, key predictors, model performance, and conclusions. | Title; Structured Abstract |
| Background and Objectives | Provides a clear rationale for developing an AI-based model specific to rural elderly women, highlighting unmet needs in LMICs and relevance to precision nutrition. Objectives and intended clinical use are explicitly stated. | Introduction (paras 1–7) |
| Source of Data | Data were derived from two datasets: (a) publicly available Singur dataset (Srimani et al., 2017) and (b) primary field data collected from Amdanga Block, West Bengal (n=458). Ethical approvals and collection protocols are described. | Methodology 2a |
| Participants | Describes inclusion/exclusion criteria, demographic characteristics, and ethical compliance for all participants. Informed consent procedures are outlined. | Methodology 2a, 2b |
| Outcome Definition | Defines cardiovascular disease (CVD) risk using composite scoring based on IDF and AHA criteria (hypertension, dyslipidemia, central obesity, impaired fasting glucose, metabolic syndrome). | Methodology 2b |
| Predictors | Lists and defines all predictors (waist circumference, blood pressure, fasting glucose, HDL, triglycerides, vitamin D), including units and clinical significance. Feature engineering expanded these to 23 derived features. | Methodology 2d, 2e |
| Sample Size and Missing Data Handling | Sample size justified through power analysis; final dataset (n=458) provided >99% statistical power. Missing data handled using KNN imputation (k=5). | Methodology 2g |
| Data Pre-processing | Outlier management (IQR capping), normalization, and class balancing via SMOTE are described in detail. | Methodology 2c |
| Model Development | Eight supervised machine learning models evaluated, including XGBoost (primary), Random Forest, Gradient Boosting, SVM, and others. Hyperparameter tuning done via Grid Search (324 combinations). | Methodology 2d |
| Model Validation | Employed stratified 5-fold cross-validation with repeated runs; performance evaluated via accuracy, precision, recall, F1-score, and AUC. | Methodology 2d; Results – Table 9 |
| Model Performance Metrics | Comprehensive reporting of accuracy (98.9%), AUC (1.000), Kappa statistics, precision, and recall. | Results – Table 9; Figure 6 |
| Model Interpretability | Feature importance analysis performed (Metabolic Score, HDL, WC as top predictors); logistic regression analysis provided interpretable validation. | Results; Discussion |
| Sensitivity and Robustness Analysis | Sensitivity analysis excluding vitamin D demonstrated <1.5% reduction in accuracy, confirming model robustness. | Discussion – “Real-World Feasibility…” |
| Risk of Bias and Overfitting Control | Class imbalance corrected via SMOTE; stratified cross-validation minimized overfitting risk. All model metrics validated on separate test data. | Methodology 2e, 2f |
| Limitations | Discusses generalizability, potential confounders, and cross-sectional design limitations. | Discussion – “Study Limitations” |
| Clinical Applicability and Use Case | Model designed for primary health center use; inputs align with NPCDCS/NFHS protocols; potential integration into telehealth and community-based screening. | Discussion – “Real-World Feasibility…” |
| Ethical and Regulatory Compliance | Ethical approval obtained from AIIH&PH, Kolkata; data anonymized and used in compliance with ethical standards. | Methodology 2l; Ethical Statement |
| Data and Code Availability | Datasets available on reasonable request; Python and R pipelines described for reproducibility. | Availability of Data and Materials |
| Funding and Conflicts of Interest | Discloses institutional support and declares absence of conflicts. | Funding; Competing Interests |
| Conclusion and Future Directions | Highlights AI-driven model’s potential for early CVD screening in underserved populations; suggests future integration with mobile health platforms. | Conclusion; Discussion (final paragraph) |

**Supplementary Table 4*:*** *Normality Assessment of Clinical Parameters Using Shapiro-Wilk Test and Implications for Statistical Analysis*

| **Parameter** | **Distribution** | **Shapiro-Wilk p-value** | **Interpretation** |
| --- | --- | --- | --- |
| HDL | Non-normal | 0.0005 | Requires non-parametric tests |
| Triglycerides | Non-normal | <0.0001 | Requires non-parametric tests |
| Fasting Blood Sugar | Non-normal | <0.0001 | Requires non-parametric tests |
| Waist Circumference | Non-normal | <0.0001 | Requires non-parametric tests |
| Systolic BP | Non-normal | <0.0001 | Requires non-parametric tests |
| Diastolic BP | Non-normal | <0.0001 | Requires non-parametric tests |
| Vitamin D | Non-normal | <0.0001 | Requires non-parametric tests |
